# When Cancer Clouds the Picture of Arterial Embolism Masquerading as Spinal Cord Compression in a Man With Metastatic Prostate Cancer

**DOI:** 10.7759/cureus.98071

**Published:** 2025-11-29

**Authors:** Abdelaziz Elsalmawy, Nadeen Woosnam, Sherif Zidan, Sam Ghaznavi

**Affiliations:** 1 Orthogeriatrics, Southend University Hospital, Southend-on-Sea, GBR; 2 Trauma and Orthopaedics, Southend University Hospital, Southend-on-Sea, GBR; 3 Trauma and Orthopaedics, Royal Free Hospital, London, GBR

**Keywords:** acute limb ischaemia, arterial embolism, diagnostic dilemma, metastatic prostate cancer, rivaroxaban

## Abstract

Acute limb pain, weakness, and sensory loss in patients with metastatic cancer often raise suspicion of spinal cord or nerve root compression; however, vascular pathologies may mimic neurological deficits, particularly in hypercoagulable malignancies. We describe a 65-year-old man with metastatic prostate cancer on full-dose Rivaroxaban who presented with a five-day history of progressive left foot pain, numbness, and weakness. Examination revealed reduced sensation over the dorsum of the foot, loss of great toe extension, weak ankle dorsiflexion, and absent distal pulses with a cold, pale foot. MRI of the lumbar spine confirmed no evidence of metastatic disease or vertebral collapse, showing only multilevel degenerative spondylosis with disc protrusions and moderate-to-severe canal stenosis. Persistent severe pain and absent pulses prompted further assessment; Doppler ultrasound demonstrated no flow below the popliteal level, and CT angiography revealed an abrupt cutoff of the left superficial femoral artery (SFA) at mid-thigh with no distal reconstitution or collateral flow, consistent with acute arterial embolism. The patient was urgently transferred to a regional vascular centre for surgical management. This case highlights the diagnostic challenge of limb symptoms in oncology patients, where neurological and vascular disorders may coexist. In metastatic prostate cancer, tumour-related hypercoagulability can cause both venous and arterial thromboses despite therapeutic anticoagulation. Awareness of this overlap, careful limb examination, and timely multimodal imaging are essential to avoid diagnostic delay and reduce morbidity.

## Introduction

Acute limb pain, weakness, or sensory disturbance in patients with metastatic malignancy often raises immediate concern for spinal cord or nerve root compression, a recognised oncologic emergency that requires prompt imaging and intervention. Prostate cancer, in particular, has a strong predilection for spinal metastases, making neurological causes a frequent diagnostic consideration when such patients present with new limb symptoms [1].

However, malignancy also induces a hypercoagulable state through tumour-mediated activation of coagulation pathways, cytokine release, and endothelial dysfunction [2]. This predisposes patients not only to venous thromboembolism (VTE) but also, more rarely, to arterial thrombotic events. The simultaneous occurrence of both venous and arterial thrombosis represents a complex and potentially life-threatening manifestation of cancer-associated coagulopathy [3,4].

The widespread adoption of direct oral anticoagulants (DOACs) such as rivaroxaban has simplified VTE management in cancer patients, yet treatment failure remains a recognised concern, particularly in advanced disease [5,6]. Breakthrough thrombotic events despite therapeutic anticoagulation highlight the limitations of DOACs in certain malignancy-related hypercoagulable states [7].

Distinguishing between neurological and vascular causes of limb weakness can be clinically challenging when symptoms overlap. Both pathologies may present with pain, numbness, and motor deficit, and in oncology patients, diagnostic momentum often favours a neurological explanation. Nonetheless, early recognition of acute limb ischaemia is critical, as delayed diagnosis can lead to irreversible tissue loss and amputation [8].

We present a diagnostically challenging case of a man with metastatic prostate cancer and known deep vein thrombosis (DVT) on full-dose rivaroxaban who developed acute arterial embolism of the same limb. This case highlights the importance of maintaining a broad differential diagnosis, performing careful vascular examination, and considering vascular imaging even when neurological causes appear more likely.

## Case presentation

A 65-year-old man with a known history of metastatic prostate cancer presented to the Emergency Department at 05:00 AM on May 17, 2025, with a five-day history of progressive left foot pain, numbness, and weakness. He described the pain as constant, worsening over time, and associated with a “dead” sensation in the foot. There was no recent trauma or fever.

Two months earlier, in March 2025, he had been diagnosed with a large proximal left lower-limb DVT extending into the external iliac vein. At that time, a contrast-enhanced CT of the thorax, abdomen, and pelvis confirmed the DVT and showed no inferior vena cava (IVC) extension. The scan also demonstrated small subpleural nodules in the right lower lobe, suggestive of possible metastatic deposits. He was commenced on full-dose rivaroxaban for anticoagulation and followed up under the oncology team.

On presentation, his vital signs were stable, and he was afebrile. Neurological examination revealed reduced sensation over the dorsum of the left foot, loss of great toe extension, and weak dorsiflexion, findings compatible with possible L5-S1 nerve root involvement. The left foot appeared cool, pale, and mildly dusky, with absent dorsalis pedis and posterior tibial pulses. Capillary refill was delayed compared to the right foot.

**Figure 1:**
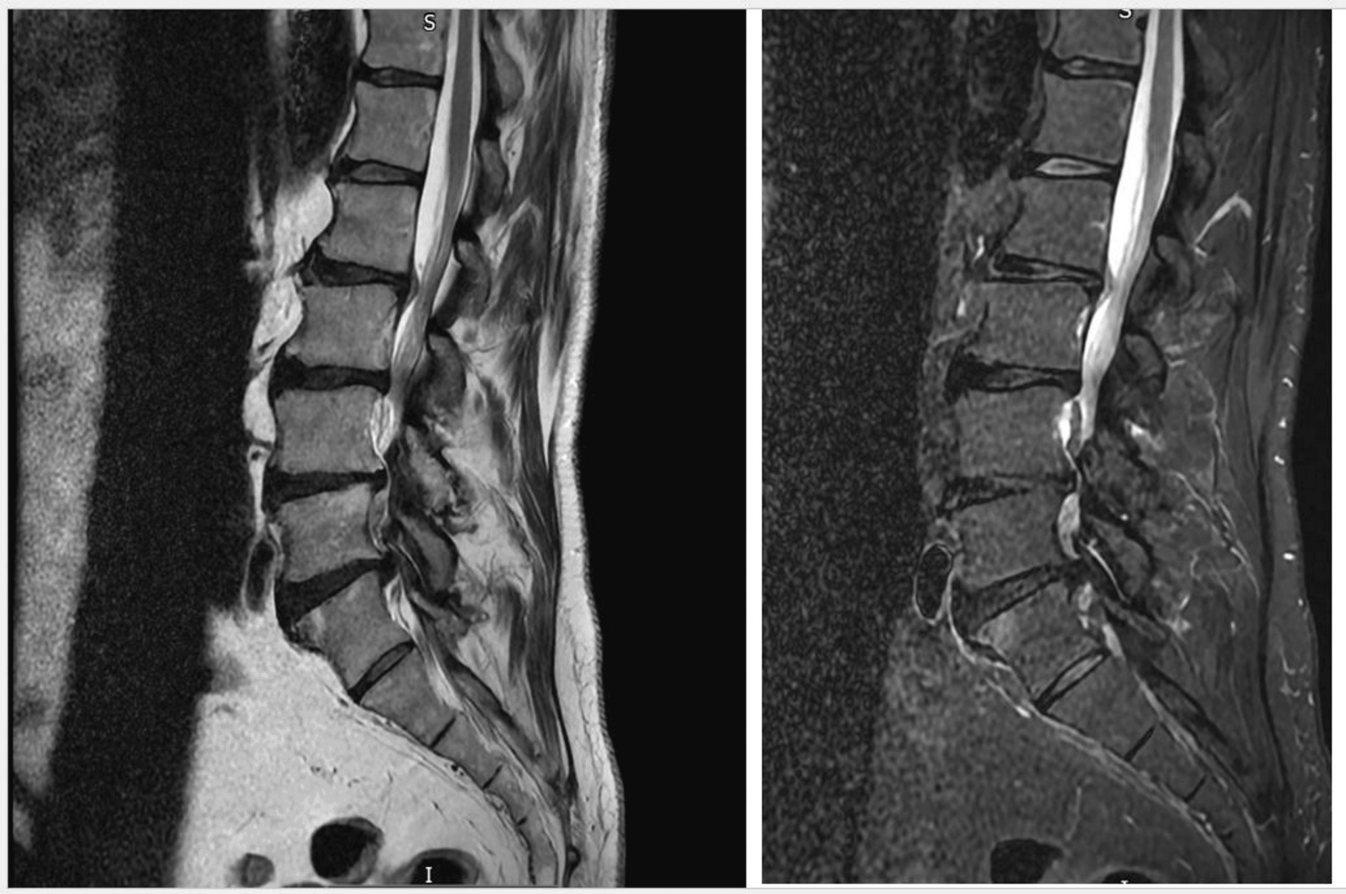
Multilevel degenerative spinal changes causing moderate-to-severe lumbar stenosis with nerve root impingement

Given his background of metastatic prostate cancer and the acute neurological deficit, there was initial concern for spinal metastasis with nerve root compression. After discussion with the orthopaedic, acute oncology, and medical teams, a lumbar spine MRI was performed. The scan revealed multilevel degenerative spondylosis with disc protrusions at L2-L3, L3-L4, and L4-L5, resulting in moderate-to-severe canal stenosis (Figure 1). Importantly, there was no evidence of vertebral metastasis, epidural mass, or spinal cord compression.

Despite these degenerative changes, the clinical features suggested an additional vascular component. The persistence of severe pain and absent pulses prompted vascular review. The bedside Doppler assessment showed no arterial signal below the popliteal level, raising concern for acute limb ischaemia. A CT angiogram of the lower limbs was subsequently arranged.

**Figure 2:**
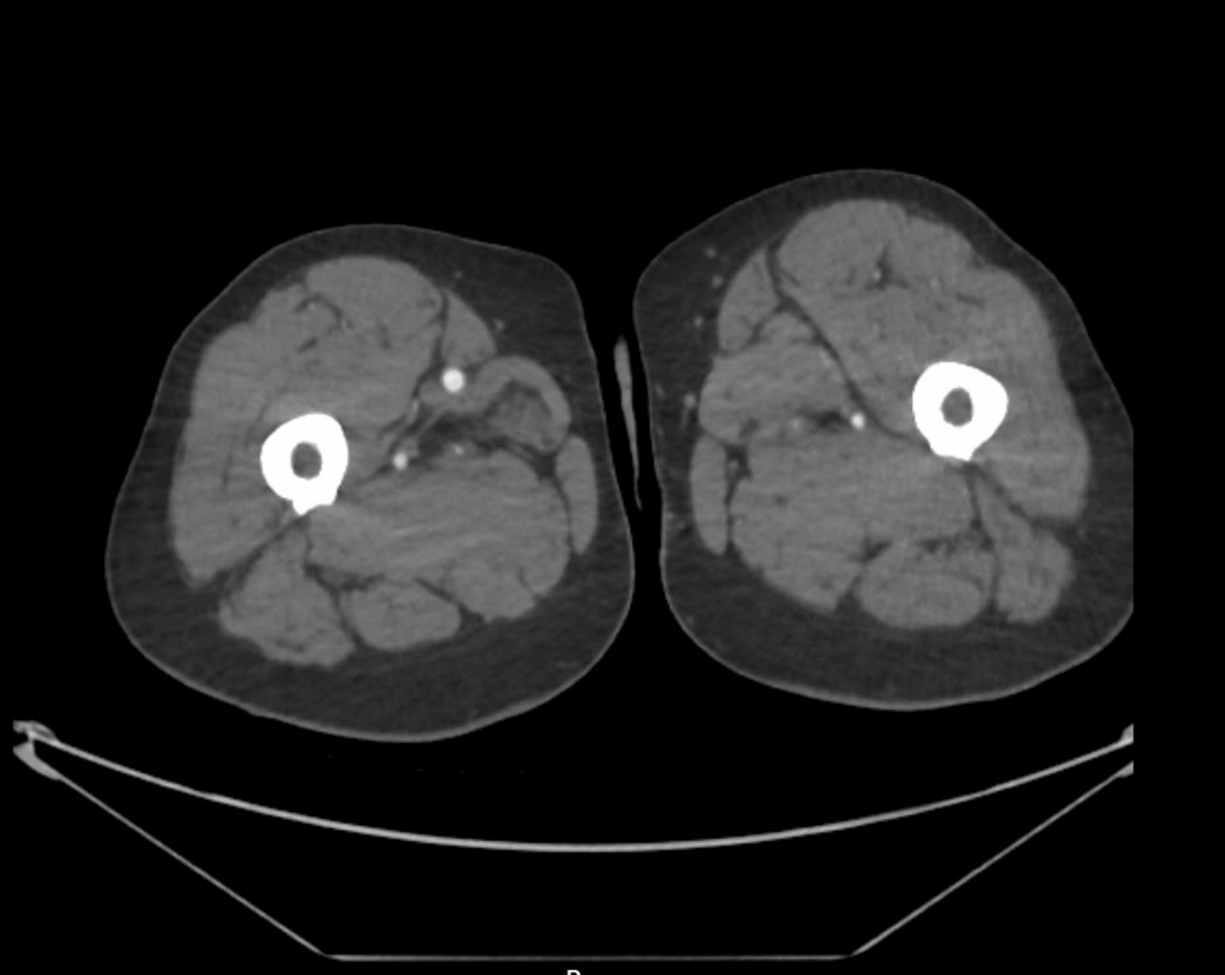
Abrupt cutoff of the left superficial femoral artery in the mid-thigh

CT angiography demonstrated an abrupt cutoff of the left superficial femoral artery (SFA) at mid-thigh level, with no distal reconstitution or collateral flow, findings consistent with acute arterial embolism (Figure 2). The contralateral lower limb showed normal arterial flow and distal runoff. The aorto-iliac vessels were patent, and no proximal thrombus was identified.

Given the confirmed diagnosis of acute limb ischaemia, the patient was transferred urgently via blue-light ambulance to the regional vascular surgery centre for definitive management.

## Discussion

This case highlights a diagnostically complex presentation in which a patient with metastatic prostate cancer and known deep vein thrombosis developed acute arterial embolism while on full-dose rivaroxaban. The combination of neurological and vascular manifestations created diagnostic ambiguity, initially suggesting lumbar nerve root compression. However, vascular evaluation revealed critical limb ischaemia due to superficial femoral artery occlusion.

Cancer-associated thrombosis and hypercoagulability

Thrombosis is a well-recognised complication of malignancy and a major cause of morbidity and mortality in cancer patients [1]. The association between cancer and thrombosis, first described by Trousseau in 1865, reflects the multifactorial activation of coagulation pathways by tumour cells, procoagulant microparticles, and inflammatory cytokines [2,3]. Prostate cancer, particularly in its metastatic stage, is strongly associated with a prothrombotic state, leading to both venous and arterial thrombotic events [4].

While VTE is common, arterial thromboembolism (ATE) in malignancy is less frequent but increasingly recognised. Population-based studies show a significantly higher incidence of arterial events, such as myocardial infarction, stroke, and limb ischaemia, within the first six months of cancer diagnosis [3,4].

Arterial embolism despite anticoagulation

The development of acute arterial occlusion despite therapeutic rivaroxaban highlights potential anticoagulation failure in the context of malignancy-induced hypercoagulability. DOACs such as apixaban, edoxaban, and rivaroxaban are now recommended for cancer-associated thrombosis [5-7]. However, real-world data indicate breakthrough events in up to 9% of patients, particularly those with advanced disease, hepatic dysfunction, or high tumour burden [5,6].

Several mechanisms may explain this failure, including excess thrombin generation that surpasses factor Xa inhibition, altered pharmacokinetics due to cancer-related cachexia, or platelet-rich thrombi that are less responsive to DOAC therapy [8,9]. Although rivaroxaban is effective for venous thrombosis, its protection against platelet-driven arterial events may be inferior to regimens combining anticoagulant and antiplatelet therapy [9,10]. Elderly cancer patients on anticoagulants also face increased risks of bleeding and recurrent thrombosis, complicating treatment decisions [11].

Diagnostic complexity and neurovascular overlap

The patient’s presentation with foot weakness and sensory loss closely mimicked L5-S1 radiculopathy, a common consequence of lumbar spondylosis or metastatic spinal disease. However, acute limb ischaemia can produce similar neurological deficits secondary to nerve hypoxia. The “six Ps” (pain, pallor, pulselessness, paraesthesia, paralysis, and poikilothermia) often evolve progressively, and early features can mislead clinicians toward neurological diagnoses [8]. A meticulous neurovascular examination remains essential in any unilateral limb deficit, particularly in oncology patients predisposed to both vascular and neurological complications.

Role of imaging in diagnostic clarification

Multimodal imaging was crucial in this case. MRI of the lumbar spine effectively excluded metastatic compression, while CT angiography, the gold standard for diagnosing acute limb ischaemia, defined the precise level of arterial occlusion and guided urgent vascular referral [12]. The coexistence of multilevel spinal stenosis and acute vascular occlusion exemplifies how dual pathology can obscure clinical reasoning, reinforcing the need for comprehensive evaluation.

Clinical implications and lessons

Cancer-associated thrombosis remains a leading cause of death and disability in oncology populations [13]. Breakthrough arterial and venous events during anticoagulation highlight the need for individualised management and close multidisciplinary collaboration between oncology, haematology, and vascular teams [14]. Clinicians must remain vigilant to the possibility of acute arterial events in patients already receiving DOAC therapy, especially when symptoms deviate from expected neurological patterns.

In summary, this case illustrates the coexistence of venous and arterial thromboses in metastatic prostate cancer and the diagnostic challenge of distinguishing vascular from neurological limb deficits. Recognition of such overlap is essential for timely intervention and improved patient outcomes.

## Conclusions

This case illustrates the diagnostic and therapeutic challenges of thromboembolic disease in cancer patients, particularly when neurological and vascular symptoms coexist. In a man with metastatic prostate cancer and known deep vein thrombosis on full-dose rivaroxaban, the development of acute arterial embolism of the same limb highlights the limitations of DOACs in the context of malignancy-induced hypercoagulability.

Clinicians should maintain a high index of suspicion for vascular compromise in oncology patients presenting with limb pain, weakness, or sensory loss, even when neurological explanations appear plausible. A meticulous neurovascular examination, prompt use of multimodal imaging, and early multidisciplinary collaboration are essential to achieve accurate diagnosis and timely intervention.

This case reinforces that not all foot drop originates from the spine and that arterial occlusion can occur despite adequate anticoagulation. Awareness of this overlap can prevent diagnostic anchoring, facilitate early recognition of acute limb ischaemia, and ultimately improve patient outcomes in complex cancer-associated thrombotic syndromes.
